# Elemental Profiling of Rice FOX Lines Leads to Characterization of a New Zn Plasma Membrane Transporter, OsZIP7

**DOI:** 10.3389/fpls.2018.00865

**Published:** 2018-07-03

**Authors:** Felipe K. Ricachenevsky, Tracy Punshon, Sichul Lee, Ben Hur N. Oliveira, Thomaz S. Trenz, Felipe dos Santos Maraschin, Maria N. Hindt, John Danku, David E. Salt, Janette P. Fett, Mary Lou Guerinot

**Affiliations:** ^1^Programa de Pós-Graduação em Biologia Celular e Molecular, Centro de Biotecnologia, Universidade Federal do Rio Grande do Sul, Porto Alegre, Brazil; ^2^Departamento de Biologia, Centro de Ciências Naturais e Exatas, Universidade Federal de Santa Maria, Santa Maria, Brazil; ^3^Department of Biological Sciences, Dartmouth College, Hanover, NH, United States; ^4^Center for Plant Aging Research, Institute for Basic Science (IBS), Daegu, South Korea; ^5^Departamento de Botânica, Universidade Federal do Rio Grande do Sul, Porto Alegre, Brazil; ^6^School of Biosciences, University of Nottingham, Loughborough, United Kingdom

**Keywords:** zinc, ZIP transporter, rice, fox lines, synchrotron x-ray fluorescence, ionomics, biofortification

## Abstract

Iron (Fe) and zinc (Zn) are essential micronutrients required for proper development in both humans and plants. Rice (*Oryza sativa* L.) grains are the staple food for nearly half of the world’s population, but a poor source of metals such as Fe and Zn. Populations that rely on milled cereals are especially prone to Fe and Zn deficiencies, the most prevalent nutritional deficiencies in humans. Biofortification is a cost-effective solution for improvement of the nutritional quality of crops. However, a better understanding of the mechanisms underlying grain accumulation of mineral nutrients is required before this approach can achieve its full potential. Characterization of gene function is more time-consuming in crops than in model species such as *Arabidopsis thaliana*. Aiming to more quickly characterize rice genes related to metal homeostasis, we applied the concept of high throughput elemental profiling (ionomics) to Arabidopsis lines heterologously expressing rice cDNAs driven by the 35S promoter, named FOX (Full Length Over-eXpressor) lines. We screened lines expressing candidate genes that could be used in the development of biofortified grain. Among the most promising candidates, we identified two lines ovexpressing the metal cation transporter *OsZIP7*. *OsZIP7* expression in Arabidopsis resulted in a 25% increase in shoot Zn concentrations compared to non-transformed plants. We further characterized OsZIP7 and showed that it is localized to the plasma membrane and is able to complement Zn transport defective (but not Fe defective) yeast mutants. Interestingly, we showed that OsZIP7 does not transport Cd, which is commonly transported by ZIP proteins. Importantly, OsZIP7-expressing lines have increased Zn concentrations in their seeds. Our results indicate that OsZIP7 is a good candidate for developing Zn biofortified rice. Moreover, we showed the use of heterologous expression of genes from crops in *A. thaliana* as a fast method for characterization of crop genes related to the ionome and potentially useful in biofortification strategies.

## Introduction

Zinc (Zn) is an essential micronutrient for plant nutrition and development, being a catalytic and structural co-factor in a large number of enzymes and regulatory proteins, including transcription factors (Marschner, 1995; Maret, 2009). However, Zn can become toxic in concentrations above a certain threshold. Fe participates in Fenton chemistry, generating reactive oxygen species. However, Zn competes with other ions for binding sites, and can become toxic in concentrations above a certain threshold (Clemens, 2001; Briat, 2002). Thus, plants have to keep Zn concentration within a narrow range for proper function. Many proteins are dedicated to Zn homeostasis, including organ and tissue partitioning as well as subcellular compartmentalization (Ricachenevsky et al., 2015).

Zn deficiency is the one the most widespread mineral nutritional disorders in humans, second only to Fe deficiency. Conservative estimates suggest that 25% of the human population is at risk of becoming Zn deficient (Maret and Sandstead, 2006). A diet composed mainly of milled cereal grains, common among poor populations, increases the risk of mineral deficiencies because staple foods, including rice (*Oryza sativa*), have low concentrations of Zn (as well as Fe) in edible tissues (Gomez-Galera et al., 2010). Biofortification, the increase of nutrient concentrations in edible portions of crops before harvesting, has been proposed as a cost-effective solution for micronutrient malnutrition (White and Broadley, 2005; Murgia et al., 2013).

Rice is a staple food for nearly half of the world’s population^1^ and a model species for monocots, making it an obvious candidate for biofortification efforts. A recent screening of a large diversity panel of rice genotypes indicated that it is possible to breed for Zn concentrations in seeds (Pinson et al., 2014). However, rice grains have lower concentration of Zn compared to other cereals, and thus genetic engineering tools might be useful to generate biofortified plants (Kennedy and Burlingame, 2003; Pfeiffer and McClafferty, 2008). In order to devise strategies for increasing micronutrient concentrations in grains, it is necessary to understand how plants acquire, distribute and store Fe and Zn within their tissues, which proteins are involved in each step and which would be good candidates for targeted, molecular breeding approaches. Despite the knowledge accumulated in recent years (Sperotto et al., 2012; Ricachenevsky et al., 2015), functional characterization of genes related to Fe and Zn homeostasis in rice is slower in comparison to the model species *Arabidopsis thaliana*. Due to genome size and complexity, and availability of protocols for genetic transformation and mutant generation, gene characterization in other crops such as wheat, maize and barley is even more time-consuming (Wu et al., 2015). Thus, strategies for fast, medium to high-throughput gene characterization would help to identify promising candidates for biofortification.

Heterologous expression of crop genes in *A. thaliana* has been used in several studies to demonstrate gene function. In order to allow high-throughput analyses of interesting phenotypes, more than 30,000 independent *A. thaliana* lines over-expressing rice genes were developed, and named FOX lines (Full-length Over-eXpressor Arabidopsis lines; (Kondou et al., 2009; Sakurai et al., 2011). These lines have been successfully used for characterization of genes associated with several processes in plants including responses to fungal and bacterial pathogens (Dubouzet et al., 2011), tolerance to abiotic stresses (Yokotani et al., 2008, 2009), enzyme characterization (Higuchi-Takeuchi et al., 2011; Anders et al., 2012) and in metabolomics profiling (Albinsky et al., 2010). More recently, similar approaches were used to describe stress-related genes in the halophyte *Eutrema salsugineum* (Ariga et al., 2015).

In this work, we characterized a collection of Arabidopsis FOX lines expressing rice genes using ionomics techniques in order to demonstrate their feasibility for the rapid functional characterization of crop genes with potential use in biofortification strategies. By combining elemental profiling by inductively-coupled plasma mass spectrometry (ICP-MS; (Salt et al., 2008) and Synchrotron X-Ray fluorescence (SXRF; Punshon et al., 2013), we described lines that express a new plasma membrane Zn transporter of the Zn-regulated, iron-regulated transporter-like protein (ZIP) family from rice, OsZIP7. Our work demonstrates that ionomics of *A. thaliana* lines heterologously expressing rice cDNAs is a useful method for the rapid characterization of genes involved in regulation of the ionome, an approach that should also be feasible for other crops.

## Materials and Methods

### Plant Materials and Growth Conditions

For ionomics profile screening, Rice FOX lines and Col-0 WT seeds were sown and cultivated as described (Lahner et al., 2003), with minor modifications. After 3 days at 4°C for stratification, trays were kept in a climate-controlled growth room with 10 h of light (90 μmol.m^-2^s^-1^)/14 h dark, humidity of 60% and temperature ranging from 19 to 22°C. Twelve plants of each genotype, including WT Col-0, were cultivated for 6 weeks, and were watered twice a week with 0.25X Hoagland solution using 10 μM Fe-HBED [N,N′-di(2-hydroxybenzyl) ethylenediamine- N,N′-diacetic acid monohydrochloride hydrate; Strem Chemicals, Inc.] as the Fe source.

For growth in axenic conditions, seeds were sterilized for 15 min in 1.5% sodium hypochloride with 0.05% SDS, washed five times in sterile H_2_O and stratified at 4°C for 3 days. Sterile 0.1% agar was used to suspend seeds, which were sown using a pipette onto plates made with full strength Gamborg’s B5 media plus vitamins, 1 mM MES [2-(*N*-morpholino)ethanesulfonic acid], 2% sucrose and 0.6% agar. After 5 days, seedlings were transferred to minimal media containing 2 mM MES, 2 mM Ca(NO_3_)_2_.4H_2_O, 0.75 mM K_2_SO_4_, 0.65 mM MgSO_4_.7H_2_O, 0.1 mM KH_2_PO_4_, 10 μM H_3_BO_3_, 0,1 μM MnSO_4_, 50 nM CuSO_4_, 5 nM (NH_4_)_6_Mo_7_O_24_ and 50 μM Fe-EDTA. ZnSO_4_ was added to a final concentration of 50 nM in control conditions, or at indicated concentrations. Seedlings were analyzed after 15 days of growth and plates were kept at 22°C with 16 h of light/8 h of dark in growth chambers.

For ICP-MS analyses of seed samples, plants were grown on soil in a growth room at 22°C with 16 h of light/8 h of dark. Seeds were collected from five plants of each genotype and analyzed by ICP-MS as above.

### Elemental Analyses by ICP-MS

Elemental concentration analyses of leaf samples were performed as described (Lahner et al., 2003), with the minor modification that the plants were grown in soil for 6 weeks. Sample handling and preparation was performed as described (Lahner et al., 2003). Data was normalized across different trays Col-0 values, which were present in each tray. All data is publicly available at www.ionomics.org for download.

For ICP-MS analyses of shoots and roots of axenically grown plants, metals were desorbed from samples for 10 min on ice with cold 5 mM CaSO_4_, 1 mM MES, pH 5.7, for 5 min on cold 5 mM CaSO_4_, 10 mM EDTA, 1 mM MES, pH 5.7 and then washed twice with cold ultrapure water (Haydon et al., 2012). Sample processing was performed as above. We used 12 replicates por line for the initial screening, five replicates per line for shoots and roots, and eight replicates for seeds ICP-MS analyses.

### Subcellular Localization

For protoplast preparation, *A. thaliana* Col-0 plants were grown in soil in a growth chamber at 22°C with 12 h of light/12 h of dark. After 4 weeks, approximately 25 leaves were detached and had their abaxial epidermis removed following digestion by the tape-sandwich method (Wu et al., 2009). Macerozyme and cellulase treatment and protoplast recovery from the remaining leaf mesophyll were performed as described (Yoo et al., 2007).

For subcellular localization in protoplasts, the OsZIP7 coding sequence lacking the stop codon was amplified using specific primers (**Supplementary Table S2**) and cloned into the pENTR/D-TOPO entry vector. Subsequently, LR recombination was performed into a vector containing a C-terminal fusion with YFP, pEarleyGate101, generating pEarleyGate101-OsZIP7. High concentrations of the final construct were prepared using PureYield^TM^ Plasmid Midiprep from Promega^®^. AtAHA2-RFP construct (Kim et al., 2001) was used as plasma membrane localization control.

Protoplast transfection was performed as described (Yoo et al., 2007). Because of the large size of the pEarleyGate101-OsZIP7 construct, 20 μg of DNA were used. For AtAHA2-RFP, 10 μg were used. For visualization of YFP and RFP signals, a Nikon Eclipse Ti inverted microscope stand was used, and image capture and processing was performed with Nikon Elements software.

*Nicotiana benthamiana* plants were grown in a growth chamber at 24° C in a 16/8 h light/dark cycles until leaves were fully expanded for agroinfiltration. Transient expression in *N. benthamiana* leaves was performed as described previously (Sparkes et al., 2006). *Agrobacterium tumefaciens* (EHA105 strain) carrying pEarleyGate101-OsZIP7 binary vector was co-infiltrated with *Agrobacterium tumefaciens* carrying pBIN20/PM-CK binary vector, which contains the coding sequence of PIP2A of *A. thaliana* fused in frame with cyan fluorescent protein (CFP; Nelson et al., 2007), in an optical density ratio of 1:1. Plasmolysis was induced using a 20% NaCl hypertonic solution. Fluorescence microscopy was performed under an Olympus FV1000 confocal laser-scanning microscope, using YFP and CFP filters. Images were captured with a high-sensitivity photomultiplier tube detector. Due to confocal microscope limitation in both co-localization experiments, we obtained subsequent images showing fluorescence signals from the same cells, without the signal overlay.

### Yeast Assays

A full-length version of OsZIP7 was amplified using specific primers (**Supplementary Table S1**) and cloned into the pDR195 vector using XhoI and BamHI sites. As a control, AtIRT1 was also amplified and cloned into pDR195 using XhoI and BamHI sites.

Yeast strains BY4743 (MATa/α his3Δ1/his3Δ1 leu2Δ0/leu2Δ0 LYS2/lys2Δ0 met15Δ0/MET15 ura3Δ0/ura3Δ0), ZHY3 (MATα *ade6 can1 his3 leu2 trp1 ura3 zrt1*::LEU2 *zrt2*::HIS3) and DEY1453 (MATa/MATα *ade2*/ADE2 *can1/can1 his3/his3 leu2/leu2 trp1/trp1 ura3/ura3 fet3-2*::HIS3/*fet3-2*::HIS3 *fet4-1*::LEU2/*fet4-1*::LEU2) were grown in YPD media pH 5.3 (DEY1453 was grown in pH 4 to increase Fe availability) and transformed with pDR195, pDR195-OsZIP7, or pDR195-AtIRT1 by the LiOAc/PEG method (Gietz and Schiestl, 2007). Selection of transformants was performed in SD media without uracil (SD –ura: 6.7 g/L yeast nitrogen base without amino acids, supplemented with 2% glucose, 0.1% casamino acids, 0.01% adenine, and 0.01% tryptophan), pH 5.3. Colonies were grown overnight in liquid SD -ura media, diluted to OD_600_ 1.0, 0.1, 0.01 and 0.001, and spotted onto plates. To test for Cd toxicity, SD -ura was amended with CdCl_2_ at given concentrations. To test for Zn deficiency, no Zn was added, and 10 μM ZnCl_2_ was added to control plates. To test for Fe deficiency, the pH was raised to 6.0, and compared to control plates at pH 5.3. Pictures were taken after 3–5 days of growth.

### Synchrotron X-Ray Fluorescence

For microtomography of seed, tomograms were collected at the bending magnet beamline X26A at the National Synchrotron Light Source, Brookhaven National Laboratory. μ-SXRF seed analyses were performed as described (Kim et al., 2006), using Col-0 and OsZIP7-FOX1 seeds deved from plants grown simultaneously. Elemental abundances (weight fraction) were calculated for the fluorescence measurements as described (McNear et al., 2005).

### Statistical Analyses

For ionomics profile comparison between FOX lines and Col-0, we used intra-tray comparisons (i.e., each line had their profile compared to Col-0 plants growing in the same tray). Concentration values for a given element (x) were considered outliers when x > Q75% + 1.5 × Q75% - Q25% or x < Q25% - 1.5 × Q75% - Q25%, where Q75% - Q25% represents 50% of the values observed (i.e., between the 1st and 3rd quartile). Statistical significance was accessed using the Wilcoxon–Mann–Whitney test and the Benjamini–Hochberg correction. All other data were subjected to ANOVA and means were compared by the Tukey HSD test.

## Results

### Rice FOX Lines Selection and Elemental Analysis

**FIGURE 1:**
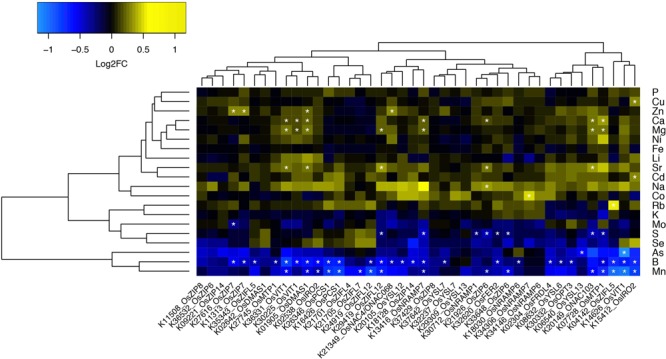
Ionomics profiling of rice FOX lines. Heatmap fold change values (on base 2 logarithm) using a color gradient from blue (low values) to yellow (high values) for each line in comparison to Col-0. Statistically significant differences are marked with an asterisk (Wilcoxon–Mann–Whitney test, *q*-value < 0.05 with Benjamini–Hochberg correction).

To perform an informed selection of Rice FOX lines, we searched the rice genome^2^ for predicted proteins with similarity to proteins described in the literature as involved in Zn and Fe homeostasis in plants. We used sequences from known genes families as queries, such as ZIP (Zinc-Regulated/Iron-Regulated Transporter Protein; Eide et al., 1996), YSL (Yellow Stripe-Like; Lee et al., 2009), ZIFL (Zinc-Induced Facilitator-Like; Haydon and Cobbett, 2007; Ricachenevsky et al., 2011), MTP (Metal Tolerance Protein; Ricachenevsky et al., 2013b), NRAMP (Natural Resistance Associated Macrophage Protein; Sasaki et al., 2012), OPT (Oligopeptide Transporter; Stacey et al., 2008), VIT (Vacuolar Iron Transporter; Zhang et al., 2012), FER (Ferritins; Stein et al., 2009), PCS (Phytochelatin Synthase; Li et al., 2007), transcription factors of the NAC (Non-Apical Meristem/Arabidopsis Transcription Activation Factor/Cup-Shaped Cotyledon) stress-related subfamily (Ricachenevsky et al., 2013a), IRO2 (Iron-related transcription factor 2; Ogo et al., 2006), and enzymes of the phytosiderophore biosynthetic pathway (Deoxymugineic acid synthase – DMAS; Bashir et al., 2006). Characterized genes for each family cited above were selected and used as queries to search the rice genome. All rice gene products showing at least 30% similarity to query sequences were compiled and used as queries to search the Rice FOX Database^3^ (Sakurai et al., 2011). We identified 42 lines expressing 24 different rice genes, comprising 13 different gene families (**Supplementary Table S1**). Fifteen genes were expressed in two or more of the FOX lines, while nine were expressed in a single line (**Figure 1**).

**FIGURE 2:**
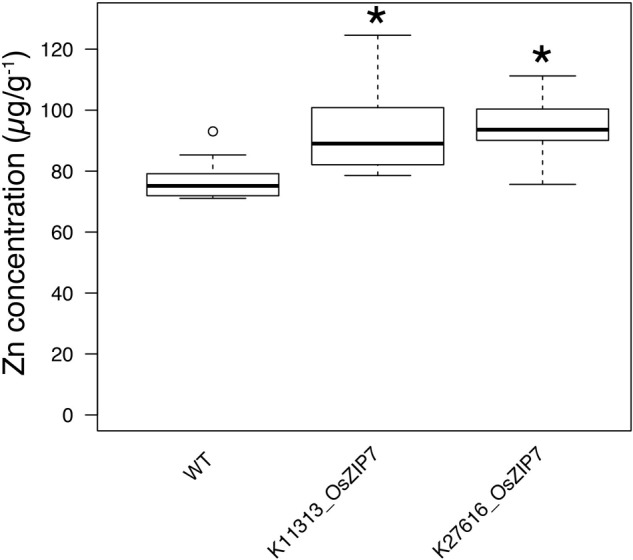
The OsZIP7-FOX lines have higher Zn concentrations in leaves. Zn concentrations of WT, K11313-OsZIP7 and K27616-OsZIP7 (two T3 lines each) were determined by ICP-MS. Black line represents the median, box edges 1st and 3rd quartile and bars minimum and maximum values. Statistically significant differences are marked with an asterisk (Wilcoxon–Mann–Whitney test, *q*-value < 0.05 with Benjamini–Hochberg correction, *n* = 12).

All FOX lines were grown under the same conditions alongside WT Col-0, in soil amended with subtoxic concentrations of trace elements, watered with Hoagland solution, and after 6 weeks leaves were collected to quantify 20 elements by ICP-MS (Lahner et al., 2003). Comparing the ionomics profiles of each FOX line with WT, we sought to find statistically significant differences in elemental concentrations (**Figure 1**). We found two lines expressing OsZIP7 that showed a consistent 25% increase in leaf Zn concentration each (**Figure 1**, lines K11313_OsZIP7 and K27616_OsZIP7). It is important to note the initial screen was performed in segregating FOX lines. We would expect changes in elemental profiles of FOX lines to be dominant, as they are a result of heterologous expression using 35S promoter. Since we analyzed 12 individual plants per line, we expected to find 3 wild types on average for each line, which would allow to detect significant changes in the ionome. Indeed, we demonstrated the feasibility of performing such a screen in FOX lines before the additional time required for isolating homozygous lines. We further confirmed the elemental profile phenotype of OsZIP7-FOX lines in the next generation (hemizygous lines; **Figure 2**) and decided to further characterize the molecular function of OsZIP7.

### OsZIP7 Can Complement Yeast Cells Defective in Zn Uptake

**FIGURE 3:**
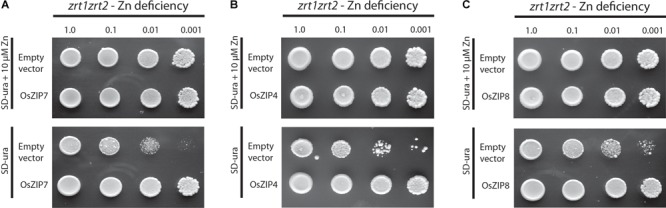
Yeast phenotype complementation assays. Empty pDR195 vector, pDR195-OsZIP7, pDR195-OsZIP4, and pDR195-OsZIP8 constructs were transformed into yeast cells. Liquid cultures were diluted as indicated before plating. OsZIP4 and OsZIP8 are positive controls **(A)** Zn-uptake defective strain *zrt1zrt2* expressing OsZIP7 growing under Zn-sufficient (10 μM Zn) or Zn-deficient (no Zn added) conditions. **(B)** Zn-uptake defective strain *zrt1zrt2* expressing OsZIP4 growing under Zn-sufficient (10 μM Zn) or Zn-deficient (no Zn added) conditions. **(C)** Zn-uptake defective strain *zrt1zrt2* expressing OsZIP8 growing under Zn-sufficient (10 μM Zn) or Zn-deficient (no Zn added) conditions.

We expressed the OsZIP7 full-length coding sequence in different yeast mutant strains to assess its metal transport ability. When introduced into the Zn uptake-defective *zrt1zrt2* mutant, OsZIP7 was able to rescue growth in low Zn medium (**Figure 3**). When expressed in the Fe uptake-defective strain *fet3fet4*, however, OsZIP7 did not restore growth in high pH medium, which lowers Fe availability (**Supplementary Figure S1**), indicating that OsZIP7 is able to transport Zn but not Fe. This is in contrast to a previous report of OsZIP7 as an Fe transporter (Yang et al., 2009). We also transformed the wild-type strain BY4743 and tested whether OsZIP7 increases cadmium (Cd) toxicity, indicative of Cd transport ability. When growing in media containing 50 μM Cd, both OsZIP7 and empty vector-transformed yeast were able to grow, while AtIRT1-transformed cells grew to a lesser extent (**Figure 3C**). Therefore, we concluded that the OsZIP7 protein is likely to function as Zn transporter, but not as an Fe or Cd transporter.

### OsZIP7 Is Localized at the Plasma Membrane in *A. thaliana* Protoplasts and *N. benthamiana* Epidermal Cells

**FIGURE 4:**
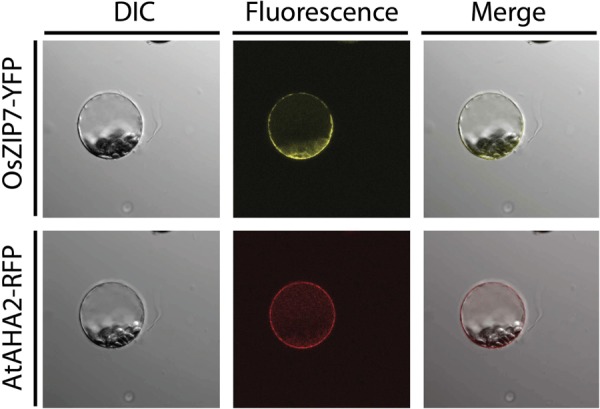
Subcellular localization of the OsZIP7-YFP construct in *A thaliana* mesophyll protoplasts after transient transformation. The plasma membrane marker protein AHA2-RFP was used as a positive control. Imaging was performed with a Nikon Eclipse Ti inverted microscope, and image capture and processing was performed with Nikon Elements software.

In order to determine the subcellular localization of OsZIP7, we transiently expressed an OsZIP7-YFP construct in *A. thaliana* protoplasts, either alone or co-transfected with AHA2-RFP, a known plasma membrane marker. The OsZIP7-YFP signal was observed in a pattern that indicated plasma membrane localization (**Figure 4**). When co-expressed with the AHA2-RFP control, (Kim et al., 2001), expression of OsZIP7-YFP and AHA2-RFP were localized in a similar pattern, although it is possible that OsZIP7 also localized to internal membranes (**Figure 4**).

**FIGURE 5:**
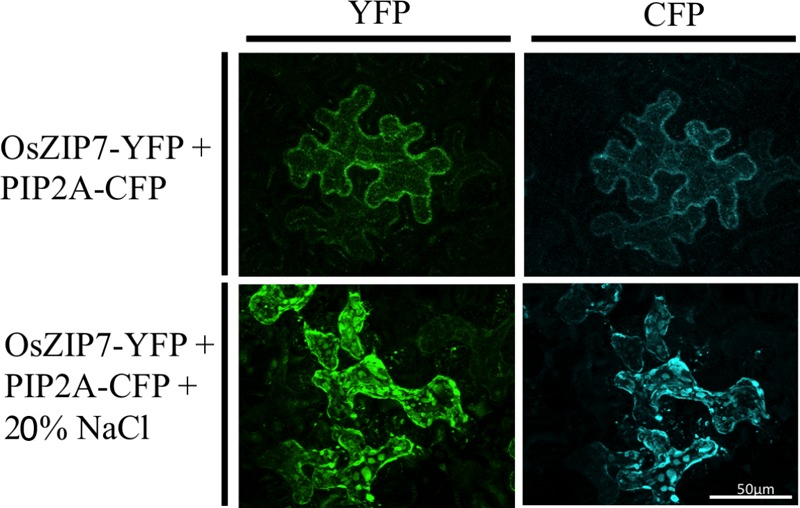
Subcellular localization of OsZIP7-YFP construct in *N. benthamiana* leaves after agroinfiltration. Plasma membrane marker PIP2A-CFP was co-infiltrated as a positive control. Plasmolysis was induced with a 20% NaCl solution.

We also transiently expressed the OsZIP7-YFP construct in *Nicotiana benthamiana* epidermal cells. *N. benthamiana* leaves were co-agroinfiltrated with the plasma membrane marker PIP2A-CFP (cyan fluorescent protein, Nelson et al., 2007). OsZIP7-YFP and PIP2A-CFP localization is highly similar in cells co-expressing both constructs (**Figure 5**). When plasmolyzed, co-localization of OsZIP-YFP with the plasma membrane marker was also evident (**Figure 5**). Plasma membrane localization is consistent with our yeast complementation results, since OsZIP7 complemented the *zrt1zrt2* mutant (**Figure 3**), which lacks two ZIP plasma membrane transporters (MacDiarmid et al., 2000). Thus, OsZIP7 is likely to be a Zn transporter localized at the plasma membrane.

### Expression of OsZIP7 in Arabidopsis Leads to Enhanced Zn Sensitivity and Disruption of Zn Root-to-Shoot Partitioning

**FIGURE 6:**
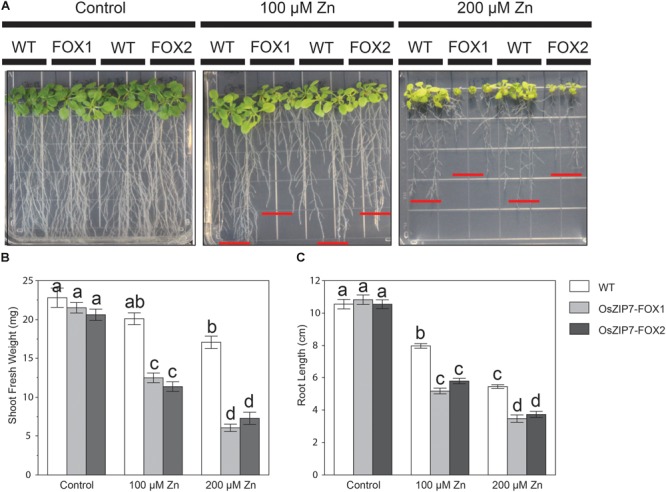
*Arabidopsis thaliana* OsZIP7-FOX lines show higher sensitivity to excessive Zn. **(A)** Seeds were sown on Gamborg’s B5 with vitamins plus 2% sucrose. After 5 days, seedlings were transferred to minimal media containing the indicated concentrations of Zn. Pictures were taken after 15 days of treatment. Shoot fresh weight **(B)** and root length **(C)** measurements were performed to quantify the changes observed. Red lines show the longest root for each line. Different letters show significant differences by ANOVA and Tukey HSD.

To gain more information on OsZIP7 function, we tested the Zn sensitivity of two independent homozygous OsZIP7-FOX lines (OsZIP7-FOX1, derived from FOX lines K11313, and OsZIP7-FOX2, derived from FOX line K27616) grown on media containing excessive Zn levels. When both OsZIP7-FOX lines were grown at control conditions, we observed similar growth compared to wild type lines (WT; **Figure 6A**). However, at 100 μM Zn, both OsZIP7-FOX lines showed decreased growth, with significantly decreased root length and shoot fresh weight compared to wild type. At 200 μM Zn, OsZIP7-FOX lines were stunted, with short roots and small shoots (**Figure 6A**). Root length was 30–35% decreased in OsZIP7-FOX lines compared to wild type in 100 and 200 μM Zn, while shoot fresh weight was about 40% decreased in 100 μM Zn and 60–65% in 200 μM Zn (**Figures 6B,C**). Thus, we concluded that OsZIP7 expression in Arabidopsis leads to increased sensitivity to Zn.

**FIGURE 7:**
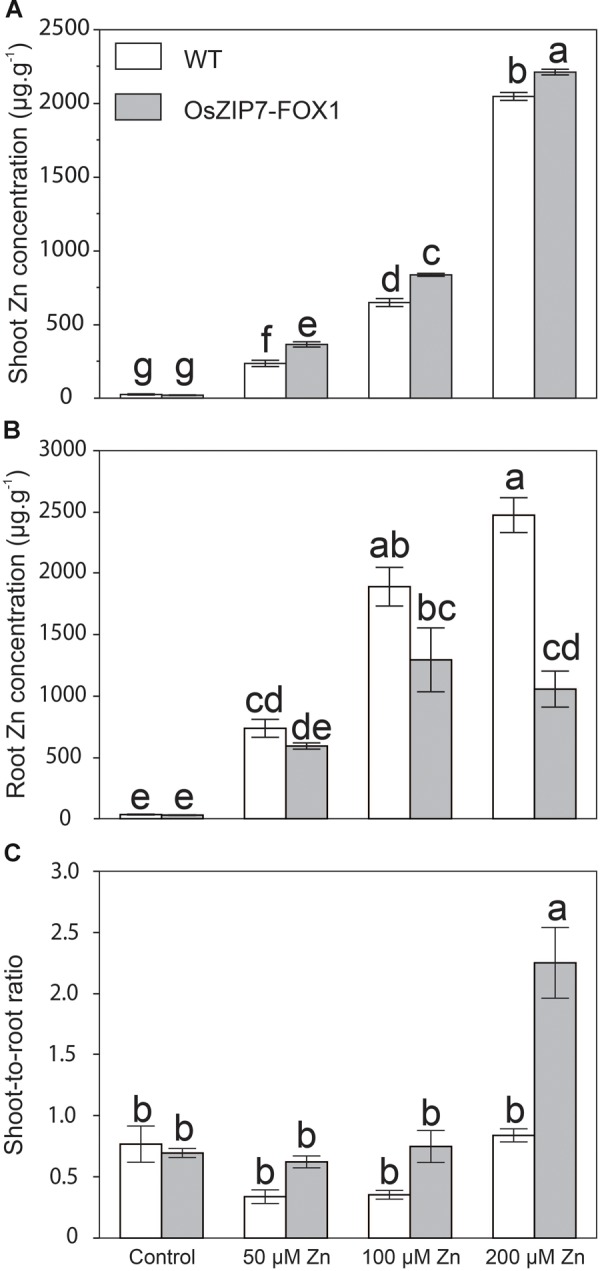
*Arabidopsis thaliana* OsZIP7-FOX lines show changes in Zn root to shoot translocation. **(A)** Zn concentration in shoots **(A)** and roots **(B)** of Col-0 and OsZIP7-FOX plants grown under control, 50, 100, or 200 μM Zn conditions (*n* = 5). **(C)** Shoot-to-root ratio of Zn concentrations. Different letters show significant differences by ANOVA and Tukey HSD.

We also quantified elemental concentration by ICP-MS in roots and shoots of wild type and OsZIP7-FOX1 plants under the same conditions, as well as in plants grown at 50 μM (the highest non-toxic Zn concentration, in our growth conditions). Zn concentrations were significantly higher in leaves of OsZIP7-FOX plants grown in media containing 50, 100, and 200 μM Zn compared to wild type (**Figure 7A**). When comparing Zn concentrations in roots of WT and OsZIP7-FOX1, the opposite effect was observed, with OsZIP7-FOX1 having lower Zn concentrations than wild type, especially under 200 μM Zn, in which OsZIP7-FOX1 root Zn concentrations were only 40% of wild type (**Figure 7B**). To clarify the change in Zn partitioning caused by expression of OsZIP7-FOX1, we compared the shoot-to-root ratio of WT and OsZIP7 plants. Clearly, ectopic expression of OsZIP7 throughout the plant led to increased root-to-shoot translocation of Zn (**Figure 7C**). Interestingly, changes in Fe concentrations were also seen in both roots and shoots of OsZIP7-FOX1 plants: roots of OsZIP7-FOX1 plants had higher Fe concentrations than in the WT when grown under 100 and 200 μM Zn, and shoots had higher Fe concentrations when grown on 200 μM Zn (**Supplementary Figure S2**).

### OsZIP7 Over-Expression Leads to Zn Accumulation in Seeds

**FIGURE 8:**
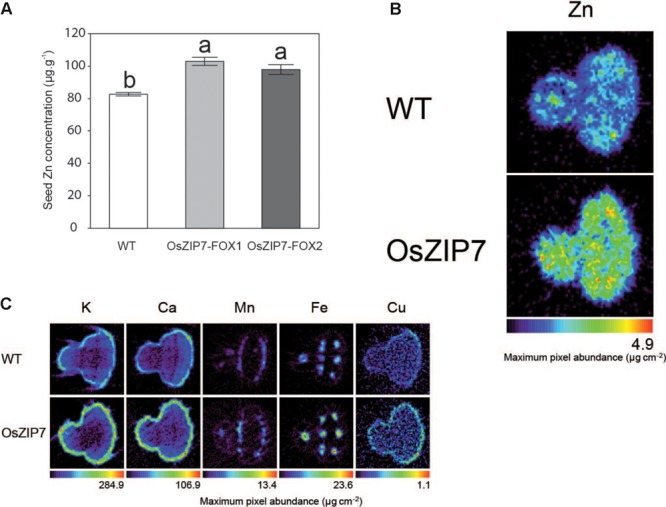
Seeds of OsZIP7-FOX1 Arabidopsis lines show higher Zn concentrations. **(A)** ICP- MS Zn concentrations in Col-0 and OsZIP7-FOX1 lines. Different letters show significant differences by ANOVA and Tukey HSD. **(B)** Zn SXRF tomogram of Col-0 and OsZIP7-FOX1. Tomograms were obtained using 250 ms dwell time, 7 μm step size, at beamline X26A at the National Synchrotron Light Source. **(C)** SXRF tomograms of Col-0 and OsZIP7-FOX1 for K, Ca, Mn, Fe, and Cu, obtained simultaneously with the Zn tomograms.

Because we are interested in good candidates for biofortification of the edible parts of plants, we decided to investigate the effect of OsZIP7 expression on Arabidopsis seed metal accumulation and distribution. We performed ICP-MS elemental quantification of WT and OsZIP7-FOX seeds from both lines. Zn concentration was 20–25% higher in the OsZIP7-FOX plants than in WT (**Figure 8A**), an increase similar to what was observed in leaves of soil-grown plants by ICP-MS (**Figure 2**). Interestingly, we also observed a small but significant decrease in Cd concentration in OsZIP7-FOX seeds, especially in OsZIP7-FOX2 line, a trait that is desirable when considering OsZIP7 as a candidate for biofortification (**Supplementary Figure S3**). The same trend was observed for Cu concentration (**Supplementary Figure S3**).

We also used synchrotron X-ray fluorescence (SXRF) microtomography to directly visualize metal distribution and abundance in seeds. Zn was clearly more abundant in OsZIP7-FOX seeds compared to WT (about twice as much), but there were no changes in distribution (**Figure 8B**). Abundance of other elements (K, Ca, Mn, Fe, and Cu) did not vary, or varied only slightly (**Figure 8C**). As we have not observed changes in these elements concentration by ICP-MS, except for OsZIP7-FOX2 line which had a slightly decrease in Cu (**Supplementary Figure S3**), it is possible that the observed differences are seed-to-seed variation. These results indicate that OsZIP7 constitutive expression increases Zn concentration in Arabidopsis seeds and slightly reduces Cd concentration, indicating OsZIP7 is a good candidate for Zn biofortification.

## Discussion

### Coupling Rice FOX Lines and Ionomics Profiling Is a Fast Method for Identification of Metal-Related Genes From Rice

Numerous proteins have been described as having a role in metal homeostasis in plants, including transporters, transcription factors and enzymes (for reviews, Hindt and Guerinot, 2012; Sinclair and Kramer, 2012; Sperotto et al., 2012; Ricachenevsky et al., 2015). However, gene characterization in crops is not as fast as in *A. thaliana*. There is need to translate the information from models to agronomically important plants. In rice, many transporters already annotated in the genome do not have an assigned molecular function, and characterization of possible targets for biofortification in other economically relevant cereals such as corn (*Zea mays*), sorghum (*Sorghum bicolor*), and wheat (*Triticum aestivum*) is difficult. Thus, the use of heterologous systems for high-throughput characterization of genes from species that are slower to cultivate or especially difficult to transform is attractive.

The use of Rice FOX lines was successful to describe proteins involved in several processes (Yokotani et al., 2008, 2009; Albinsky et al., 2010; Dubouzet et al., 2011; Higuchi-Takeuchi et al., 2011; Anders et al., 2012). In this work, we highlight the feasibility of using FOX lines coupled with ionomics profiling for characterization of metal-related genes from crop species, such as rice. Besides OsZIP7, which we discussed in detail, our screen identifies other examples of interesting lines that might be studied in depth to understand their role in the regulation of the ionome.

The results reported here are derived from a subset of FOX lines selected because they contain cDNAs from gene families involved in metal homeostasis. Similar focused approaches have successfully identified genes from *E. salsugineum* that confer heat or heat and salt stress tolerance when expressed in *A. thaliana*, in which 78 and 433 lines were tested, respectively (Higashi et al., 2013; Ariga et al., 2015). In these studies, T2 generations were also screened for stress tolerance. Thus, it is clear that phenotyping of FOX lines and similar tools can be performed even without isolation of homozygous lines, since it is expected that altered phenotypes would be dominant. It should be considered that an unbiased screen (i.e., not focused on selected metal transporters) lines expressing heterologous genes could lead to the identification of previously unknown regulators of the ionome. Moreover, it should also be noted that constitutive expression of a gene in a heterologous system might overcome regulatory mechanisms that might modulate protein activity (i.e., post-transcriptional regulation) in their native environment, increasing the chances of identifying interesting genes that could otherwise be regulated by transcriptional and post-transcriptional mechanisms.

### OsZIP7 Is a New Zn Transporter

The first member of the ZIP (Zinc-regulated/Iron-Regulated Protein) family of transporters described was AtIRT1 (Eide et al., 1996), followed by characterization of several ZIP members in Arabidopsis, rice, corn, barley (*Hordeum vulgare*) among other species (Lee et al., 2010a,b; Li et al., 2013; Milner et al., 2013; Tiong et al., 2014). Plants harbor many ZIP genes in their genomes, with as many as 16 *loci* in the genomes of some Poaceae (Tiong et al., 2015). ZIP transporters are known for having broad substrate specificity: AtIRT1 is able to transport Zn^+2^, Fe^2+^, Mn^2+^, Cd^2+^, Co^2+^, Ni^2+^, and Fe^3+^ (Korshunova et al., 1999) while its rice ortholog OsIRT1 transports Fe^2+^, Zn^2+^, and Cd^2+^ (Ishimaru et al., 2006; Lee and An, 2009). AtIRT2 and AtIRT3 transport Fe^2+^ and Zn^+2^, but not Mn^2+^ or Cd^2+^ (Vert et al., 2001; Lin et al., 2009). In Arabidopsis, others ZIPs are commonly Zn^2+^ or Zn^2+^ and Mn^2+^ transporters, with AtZIP7 also being able to transport Fe^2+^ (Milner et al., 2013). Moreover, ZIP proteins characterized in plants are mostly localized to the plasma membrane, which also seems to be true for OsZIP7 based on our data (**Figures 4, 5**). *OsZIP7* is the closest rice homolog of barley *HvZIP7* and maize *ZmZIP7* (Tiong et al., 2014; Li et al., 2016) and Arabidopsis *AtIRT3* and *AtZIP4* (Li et al., 2013; Tiong et al., 2015). Of these, HvZIP7 and AtIRT3 had their subcellular localization determined to be at the plasma membrane (Lin et al., 2009; Tiong et al., 2014).

Here we have shown that OsZIP7 was able to complement the Zn-deficient *zrt1zrt2* yeast mutant, but not the Fe-deficient *fet3fet4* (**Figure 3**). *OsZIP7* has been indicated as the rice ortholog of barley *HvZIP7*, which was recently characterized as a Zn transporter (Tiong et al., 2014, 2015). HvZIP7 was localized to the plasma membrane and increased plant Zn root-to-shoot translocation when compared to WT controls in over-expressing barley plants (Tiong et al., 2014). This is consistent with our observation that OsZIP7 expression in *A. thaliana* under the control of 35S promoter led to increased Zn concentrations in leaves and seeds and increased root-to-shoot Zn translocation (**Figures 7, 8**).

Expression of OsZIP7 in Arabidopsis led to increased root-to-shoot Zn translocation when plants are exposed to high Zn in the growth media, with roots of OsZIP7-FOX lines showing lower Zn concentrations compared to WT, whereas shoots have increased Zn concentrations (**Figure 7**). This is similar to what was observed for HvZIP7 over-expression in barley, with plants showing higher Zn concentration in shoots and lower in roots compared to null-segregant lines (Tiong et al., 2014). In both our OsZIP7-FOX lines and in HvZIP7 over-expressing plants, Zn concentrations in shoots and leaves were not changed under control conditions. One possible explanation for these phenotypes is that OsZIP7 expression might increase sink strength in shoots, while also causing increased primary Zn uptake in shoots. Zn xylem-loading transporters such as AtHMA2/AtHMA4 (Hussain et al., 2004) may not limit Zn translocation to shoots under such conditions. Although the precise mechanism is not clear, OsZIP7 expression in Arabidopsis and HvZIP7 over-expression in barley seem to result in distinct phenotypes compared to over-expression of other ZIP transporters such as OsZIP4, OsZIP5, and OsZIP8 (Ishimaru et al., 2007; Lee et al., 2010a,b). In seeds, however, OsZIP7-FOX lines and HvZIP7 over-expressing lines showed increased Zn concentrations even without excessive Zn in the media, indicating that higher Zn accumulation for biofortification using OsZIP7/HvZIP7 may not require Zn addition (**Figure 8**, Tiong et al., 2014).

Interestingly, two OsZIP7 protein sequences have been reported in rice, differing in only four amino acid positions: OsZIP7, which is characterized in this work, and OsZIP7a, characterized by Yang et al. (2009). Three aminoacid changes are in positions outside transmembrane domains, while one is inside the VII domain. OsZIP7a was shown to not complement Zn-defective yeast mutants (Yang et al., 2009). HvZIP7 failed to complement the yeast strain *zrt1zrt2*, although several other lines of evidence indicate its function as a Zn transporter (Tiong et al., 2014). Here we showed that OsZIP7 is able to rescue the *zrt1zrt2* yeast mutant phenotype to some extent (**Figure 3**). Considering the increased Zn sensitivity of Arabidopsis expressing OsZIP7 (**Figure 6**), these results suggest that OsZIP7 is a Zn transporter. It is possible that OsZIP7 is a low-affinity Zn transporter, as suggested for its closest homologous gene from barley (HvZIP7; Tiong et al., 2014). Interestingly, OsZIP7a has been described as able to complement *fet3fet4*, suggesting it could transport Fe (Yang et al., 2009). We did not observe *fet3fet4* complementation when *fet3fet4*-expressing OsZIP7 was cultivated in high pH media, while AtIRT1-expressing yeast was able to grow (**Figure 3**), indicating that OsZIP7 does not transport Fe. However, it is still possible that OsZIP7 transports Fe. One hypothesis is that transport is dependent on pH, with high pH decreasing transport function. Despite that, our results support that OsZIP7 is a Zn transporter.

The FOX lines expressing OsZIP7 showed increased Fe concentrations in roots and shoots upon high Zn concentration in the growth media (**Supplementary Figure S2**). This may indicate that OsZIP7 might transport Fe, although it is not clear why Fe concentrations would increase only under high Zn. Heterologous expression in Arabidopsis of OsZIP7 maize ortholog, ZmZIP7, led to increased Fe and Zn concentrations in all tissues, and concomitant up-regulation of the Fe uptake regulon, including AtIRT1 (Li et al., 2016). Conversely, HvZIP7 over-expression in barley does not change Fe concentrations in either shoots or roots, even when plants are cultivated under high Zn in the growth media (Tiong et al., 2014). A possible explanation is that high Zn concentrations induced Fe-deficiency and Fe uptake genes, leading to increased root and shoot Fe concentrations (**Supplementary Figure S2**). Thus, it is more likely that OsZIP7 is not able transport Fe. Still, future work should address if OsZIP7 and OsZIP7a differ in their substrates and if the four distinct aminoacids can change metal specificity (Yang et al., 2009).

### Potential of OsZIP7 for Zn Biofortification of Rice Seeds

OsZIP7 expression in Arabidopsis increased Zn concentration in seeds by 25% (**Figure 8**). Similarly, HvZIP7 over-expression in barley led to significant increase in Zn concentration in grains, with no changes in other elements (Tiong et al., 2014), whereas expression of ZmZIP7 in Arabidopsis led to increased Zn and Fe concentrations in seeds. Interestingly, we have also observed a decrease in Cd concentration of 12–24% in seeds (**Supplementary Figure S3**), indicating that OsZIP7 over-expression in rice could lead to increase Zn in grains without concomitantly increasing Cd levels. From a biofortification perspective, that makes OsZIP7 a good candidate for genetic engineering, since Cd co-transport when manipulating Zn and Fe transport such as the ZIP family members should be considered (Slamet-Loedin et al., 2015). OsZIP7 is highly expressed in developing grains in rice plants (**Supplementary Figure S4**).

Several different genes have been used to improve Zn concentration in rice grains, and increases have been moderate so far (for a review, see Ricachenevsky et al., 2015). Two successful transgenic approaches involved activation tagging or over-expression of nicotianamine synthase (NAS) genes (Johnson et al., 2011; Lee et al., 2011). Presumably, increased levels of nicotianamine in these plants facilitate Zn loading in the phloem and translocation to grains, but increased available Zn for translocation might lead to further accumulation. Thus, there is still potential to increase Zn levels. Either OsZIP7 over-expression as a single transgene, combined with OsNAS2 of expressed in specific cell types such as the endosperm could be promising to generate biofortified rice in the future.

## Conclusion

We have demonstrated that Arabidopsis lines generated to heterologously express rice genes are useful for fast screening genes that are involved in metal homeostasis when combined with elemental analyses. We have also molecularly characterized OsZIP7, a Zn plasma membrane-localized transporter from rice. Based on our results, OsZIP7 is a good candidate for over-expression in rice to generate lines that are able to accumulate Zn in their seeds.

## Author Contributions

FKR, TP, DES, JPF, and MLG designed the experiments. FKR, TP, and MNH performed the experiments. FKR, TP, BHNO, and JD performed the analyses. FKR, TP, SL, BHNO, TST, FSM, MNH, DES, JPF, and MLG wrote the manuscript. All authors approved the manuscript.

## Conflict of Interest Statement

The authors declare that the research was conducted in the absence of any commercial or financial relationships that could be construed as a potential conflict of interest.

## Footnotes

1 http://www.fao.org/rice2004/en/rice-us.htm

2 http://rice.plantbiology.msu.edu/

3 http://ricefox.psc.riken.jp/

## Supplementary Material

FIGURE S1 Yeast phenotype complementation assays. Empty pDR195 vector, pDR195-OsZIP7 or pDR195-AtIRT1 constructs were transformed into yeast cells. Liquid cultures were diluted as indicated before plating. **(A)** Fe-uptake defective strain *fet3fet4* transformed with each construct growing under Fe-sufficient (pH 5.3) or Fe-deficient (pH 6.0) conditions. **(B)** Wild type strain BY4743 transformed with each construct growing under control or 50 μM Cd conditions.

FIGURE S2 Metal concentrations in shoots **(A–D)** and roots **(E–H)** of Col-0 (white bars) and OsZIP7-FOX Arabidopsis plants (gray bars) grown for 15 days in Minimal Media containing 50, 100, or 200 μM Zn (*n* = 5). Concentrations of Mn **(A,E)**, Fe **(B,F)**, Cu **(C,G)**, and Cd **(D,H)** are shown. Different letters show significant differences by ANOVA and Tukey HSD.

FIGURE S3 Metal concentrations in seeds of Col-0 and OsZIP7-FOX1 and OsZIP7-FOX2 plants (*n* = 8). Concentrations of Mn **(A)**, Fe **(B)**, Cu **(C)**, and Cd **(D)** are shown. Different letters show significant differences by ANOVA and Tukey HSD.

FIGURE S4 Rice OsZIP7 transporter expression pattern based on data from the eFP Browser public database (http://bar.utoronto.ca/efp_rice/cgi-bin/efpWeb.cgi).

TABLE S1 Rice FOX lines used in this work.

TABLE S2 Primers used in this work.
