# Supplementary figures and images for: Elemental Profiling of Rice FOX Lines Leads to Characterization of a New Zn Plasma Membrane Transporter, OsZIP7
Source: Front Plant Sci. 2018 Jul 3;9:865. doi: 10.3389/fpls.2018.00865 (PMC6037872; doi:10.3389/fpls.2018.00865)

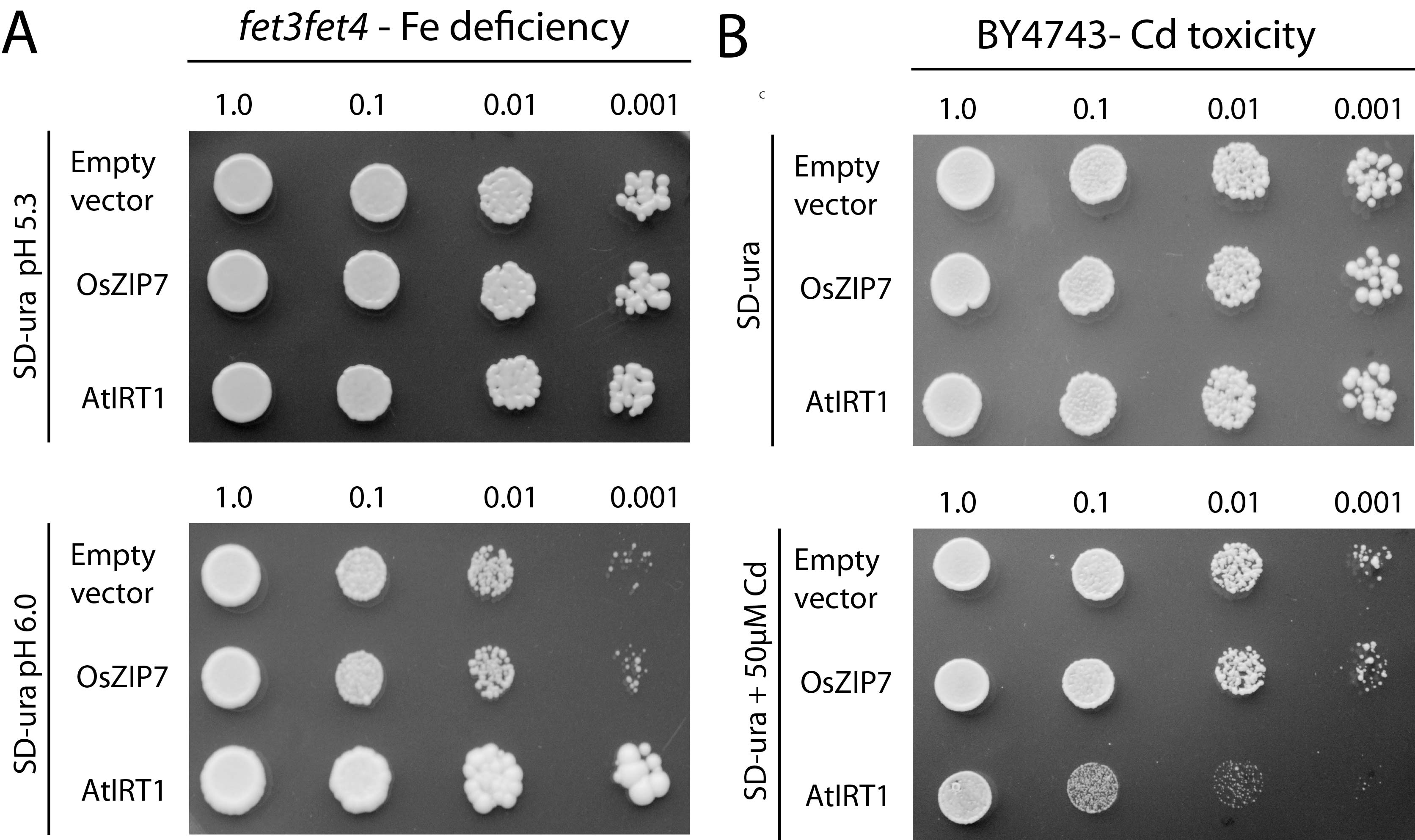

Supplement: FIGURE S1 — Yeast phenotype complementation assays. Empty pDR195 vector, pDR195-OsZIP7 or pDR195-AtIRT1 constructs were transformed into yeast cells. Liquid cultures were diluted as indicated before plating. (A) Fe-uptake defective strain fet3fet4 transformed with each construct growing under Fe-sufficient (pH 5.3) or Fe-deficient (pH 6.0) conditions. (B) Wild type strain BY4743 transformed with each construct growing under control or 50 μM Cd conditions. [file Image_1.JPEG]

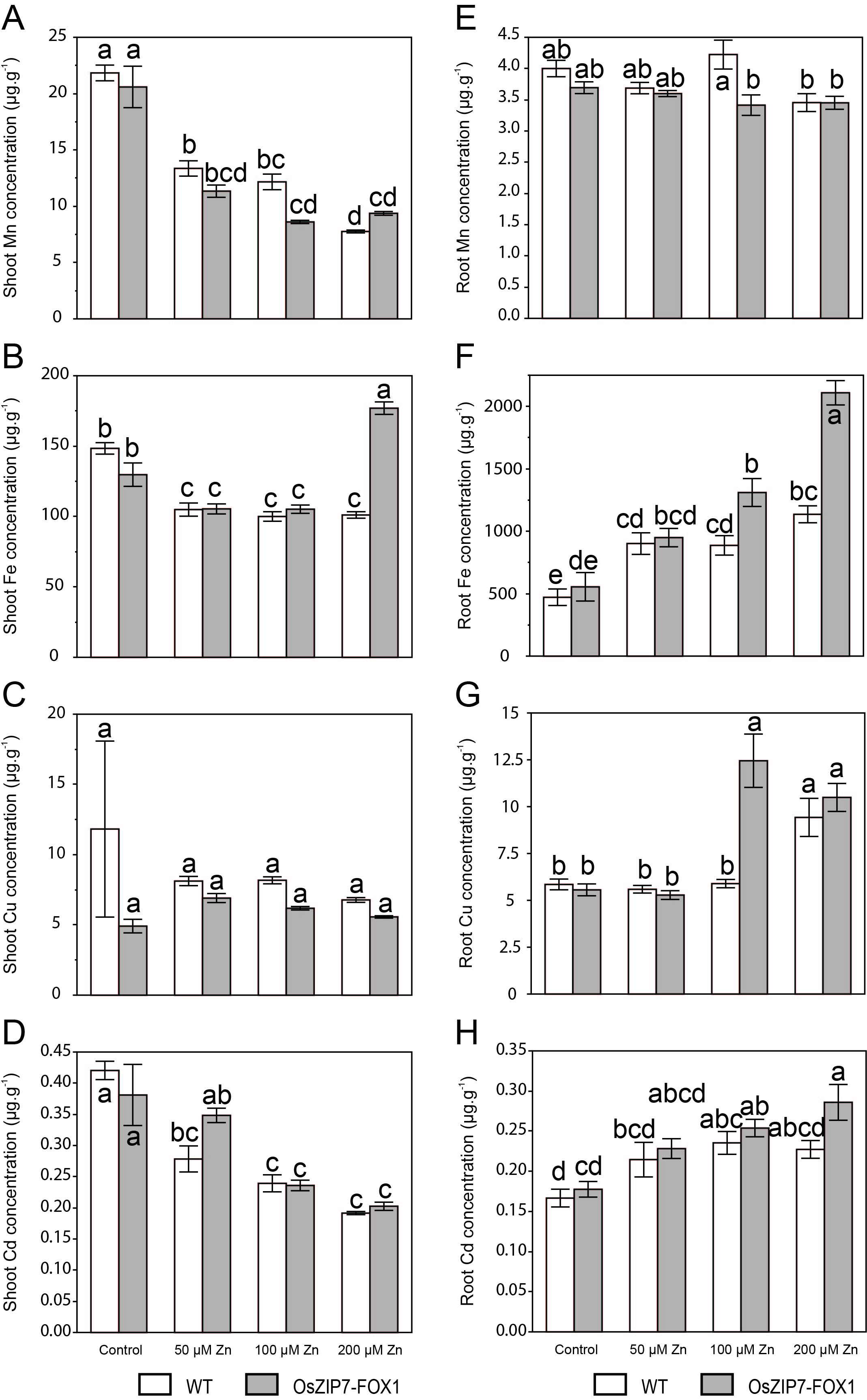

Supplement: FIGURE S2 — Metal concentrations in shoots (A–D) and roots (E–H) of Col-0 (white bars) and OsZIP7-FOX Arabidopsis plants (gray bars) grown for 15 days in Minimal Media containing 50, 100, or 200 μM Zn (n = 5). Concentrations of Mn (A,E), Fe (B,F), Cu (C,G), and Cd (D,H) are shown. Different letters show significant differences by ANOVA and Tukey HSD. [file Image_2.JPEG]

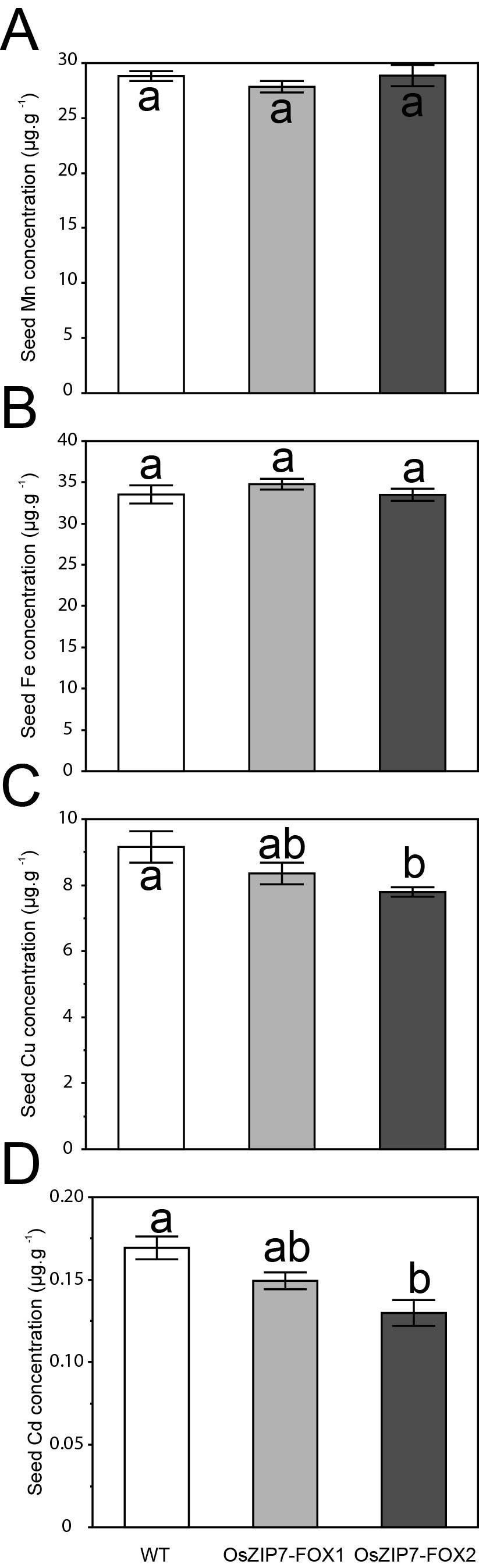

Supplement: FIGURE S3 — Metal concentrations in seeds of Col-0 and OsZIP7-FOX1 and OsZIP7-FOX2 plants (n = 8). Concentrations of Mn (A), Fe (B), Cu (C), and Cd (D) are shown. Different letters show significant differences by ANOVA and Tukey HSD. [file Image_3.JPEG]

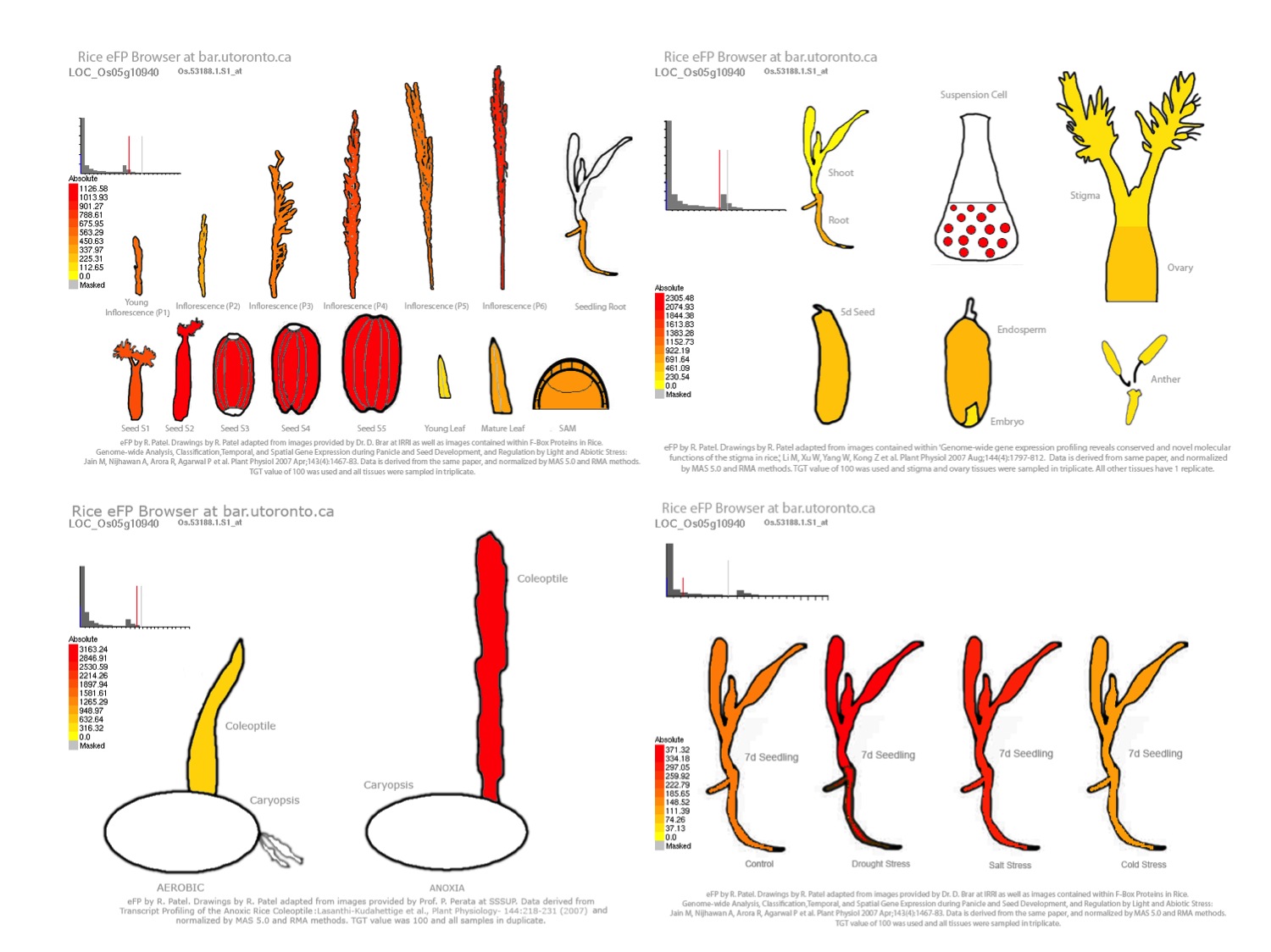

Supplement: FIGURE S4 — Rice OsZIP7 transporter expression pattern based on data from the eFP Browser public database (http://bar.utoronto.ca/efp_rice/cgi-bin/efpWeb.cgi). [file Image_4.JPEG]
