# Supplementary material for: Elemental Profiling of Rice FOX Lines Leads to Characterization of a New Zn Plasma Membrane Transporter, OsZIP7
Source: Front Plant Sci. 2018 Jul 3;9:865. doi: 10.3389/fpls.2018.00865 (PMC6037872; doi:10.3389/fpls.2018.00865)
Supplement: TABLE S1 — Rice FOX lines used in this work. [file Table_1.DOCX]

**Supplemental Table 1.** Rice FOX lines used in this work.

| Number | Line Number | Gene | KOME cDNA Number | Locus Number |
| --- | --- | --- | --- | --- |
| 1 | K02538 | OsIRO2 | J023035P08 | LOC_Os01g72370 |
| 2 | K15412 | OsIRO2 | J033041D19 | LOC_Os01g72370 |
| 3 | K02642 | OsDMAS1 | J033064L19 | LOC_Os03g13390 |
| 4 | K01905 | OsDMAS1 | J033064L19 | LOC_Os03g13390 |
| 5 | K14626 | OsVIT2 | J023101O19 | LOC_Os09g23300 |
| 6 | K30225 | OsVIT2 | J023101O19 | LOC_Os09g23300 |
| 7 | K36331 | OsVIT2 | J023101O19 | LOC_Os09g23300 |
| 8 | K32620 | OsFER2 | J033088E01 | LOC_Os12g01530 |
| 9 | K30712 | OsNRAMP1 | J033132H19 | LOC_Os07g15460 |
| 10 | K18030 | OsNRAMP6 | J013089K10 | LOC_Os01g31870 |
| 11 | K34146 | OsNRAMP6 | J013164K10 | LOC_Os01g31870 |
| 12 | K13416 | OsNRAMP7 | J023095G02 | LOC_Os12g39180 |
| 13 | K34306 | OsNRAMP7 | J023095G02 | LOC_Os12g39180 |
| 14 | K21926 | OsZIP6 | J033138E20 | LOC_Os05g07210 |
| 15 | K33648 | OsZIP6 | J033138E20 | LOC_Os05g07210 |
| 16 | K36532 | OsZIP6 | J033138E20 | LOC_Os05g07210 |
| 17 | K11313 | OsZIP7 | J023087M13 | LOC_Os05g10940 |
| 18 | K27616 | OsZIP7 | J023087M13 | LOC_Os05g10940 |
| 19 | K11508 | OsZIP8 | J013157C18 | LOC_Os02g10230 |
| 20 | K37429 | OsZIP8 | J013157C18 | LOC_Os02g10230 |
| 21 | K09221 | OsZIP14 | J033089E10 | LOC_Os08g36420 |
| 22 | K18128 | OsZIP14 | J033089E10 | LOC_Os08g36420 |
| 23 | K08632 | OsYSL6 | J013070E15 | LOC_Os04g32050 |
| 24 | K32237 | OsYSL7 | J023003B17 | LOC_Os02g02450 |
| 25 | K37642 | OsYSL7 | J023003B17 | LOC_Os02g02450 |
| 26 | K20105 | OsYSL12 | J023023O07 | LOC_Os04g44320 |
| 27 | K29309 | OsYSL13 | J013096O22 | LOC_Os04g44300 |
| 28 | K06540 | OsYSL13 | J013096O22 | LOC_Os04g44300 |
| 29 | K02304 | OsFRDL4 | J013001B22 | LOC_Os12g01580 |
| 30 | K16426 | OsPCS1 | J013081H24 | LOC_Os06g01260 |
| 31 | K26346 | OsPCS1 | J023112B16 | LOC_Os06g01260 |
| 32 | K21701 | OsZIFL4 | J023019M18 | LOC_Os11g04020 |
| 33 | K35343 | OsZIFL5 | J013072J11 | LOC_Os11g04030 |
| 34 | K04142 | OsZIFL5 | J013072J11 | LOC_Os11g04030 |
| 35 | K21705 | OsZIFL7 | J023109G16 | LOC_Os11g04104 |
| 36 | K24919 | OsZIFL12 | J033105E18 | LOC_Os12g03899 |
| 37 | K29419 | OsZIFL12 | J033105E18 | LOC_Os12g03899 |
| 38 | K30332 | OsOPT3 | J013116J20 | LOC_Os06g03560 |
| 39 | K07130 | OsMTP1 | J023117P18 | LOC_Os05g03780 |
| 40 | K07728 | OsMTP1 | J023117P18 | LOC_Os05g03780 |
| 41 | K27745 | OsMTP1 | J023117P18 | LOC_Os05g03780 |
| 42 | K20149 | ONAC103 | J013157G23 | LOC_Os07g48450 |
