# Supplementary material for: Elemental Profiling of Rice FOX Lines Leads to Characterization of a New Zn Plasma Membrane Transporter, OsZIP7
Source: Front Plant Sci. 2018 Jul 3;9:865. doi: 10.3389/fpls.2018.00865 (PMC6037872; doi:10.3389/fpls.2018.00865)
Supplement: TABLE S2 — Primers used in this work. [file Table_2.DOCX]

**Supplemental Table 2.** Primers used in this work.

| Primer name | Sequence 5’ to 3’ | Purpose |
| --- | --- | --- |
| AtIRT1_ XhoI_F | AATCTCGAGATGAAAACAATCTTCCTCGTACTCA | Cloning AtIRT1 in pDR195 |
| AtIRT1_BamHI_R | AATGGATCCTTAAGCCCATTTGGCGATAA | Cloning AtIRT1 in pDR195 |
| OsZIP7_XhoI_F | AATCTCGAGATGGAGCGGTTCGTGCAGT | Cloning OsZIP7 in pDR195 |
| OsZIP7_BamHI_R | AATGGATCCTCAGGCCCAGATTGCAAG | Cloning OsZIP7 in pDR195 |
| OsZIP7_pENTR_F | CACCATGGAGCGGTTCGTGCAGT | Cloning OsZIP7 in pENTR |
| OsZIP7_pENTR_NS_R | GGCCCAGATTGCAAGGGAT | Cloning OsZIP7 in pENTR |
| OsZIP7_Nterm_F | GGTTCGTGCAGTTCTTGAGG | Expression in FOX lines |
| OsZIP7_Nterm_R | GTGCAGCATGTGGACGAA | Expression in FOX lines |
| OsZIP7_Cterm_F | CTCTAGTGGTGGAGGGCATC | Expression in FOX lines |
| OsZIP7_Cterm_R | GCAAGGGATGACATGGAGAG | Expression in FOX lines |
